# Geraniol alleviates DNCB-induced atopic dermatitis in mice by downregulating IL-4/IL-13 and reducing inflammation

**DOI:** 10.17305/bb.2026.13992

**Published:** 2026-03-10

**Authors:** Hafsa Nasr, Arham Shabbir, Rabbia Kalim, Aisha Mobashar, Ali F Almutairy, Hamoud Alotaibi, Saleh Alfuraih, Abdulaziz H Alanazi, Sulaiman Mohammed Abdullah Alnasser, Waad Alrohily, Latifah Al Shammari, Tabinda Fatima, Ashfaq Ahmad

**Affiliations:** 1 Department of Pharmacology, Institute of Pharmacy, Faculty of Pharmaceutical and Allied Health Sciences, Lahore College for Women University, Lahore, Pakistan; 2 Faculty of Pharmacy, The University of Lahore, Lahore, Pakistan; 3 Department of Pharmacology and Toxicology, College of Pharmacy, Qassim University, Buraydah, Saudi Arabia; 4 Department of Pharmaceutics, Faculty of Pharmacy, Northern Border University, Rafha, Saudi Arabia; 5 Department of Pharmacology and Toxicology, College of Pharmacy, Northern Border University, Rafha, Saudi Arabia; 6 Department of Clinical Practice, College of Pharmacy, Northern Border University, Rafha, Saudi Arabia; 7 Department of Pharmacy Practice, College of Pharmacy, Taibah University, Medina, Saudi Arabia; 8 Department of Pharmaceutical Chemistry, College of Pharmacy, University of Hafr Al Batin, Hafr Al Batin, Saudi Arabia; 9 Department of Pharmacy Practice, College of Pharmacy, University of Hafr Al Batin, Hafr Al Batin, Saudi Arabia

**Keywords:** Atopic dermatitis, geraniol, inflammation, interleukin-4, molecular docking.

## Abstract

Atopic dermatitis (AD) is a chronic inflammatory skin condition characterized by recurrent itching, predominantly affecting children but also impacting adults. Geraniol, a monoterpene alcohol found in various aromatic plant-based essential oils, possesses a pleasant rose-like scent. This study aimed to investigate the therapeutic potential of geraniol in a mouse model of atopic dermatitis by elucidating its anti-inflammatory and immunomodulatory properties. Mice were subjected to 2% 2,4-Dinitrochlorobenzene (DNCB) to induce AD, and treated with both oral and topical administrations of prednisolone and geraniol from day 7 to day 19. Macroscopic assessments of ear and dorsal skin, as well as ear thickness, were conducted on days 0, 7, and 19. Total leukocyte count (TLC) and differential leukocyte count (DLC) were measured in blood samples using an automatic hematology analyzer. Ear tissues were analyzed for mRNA expression levels of IL-4 and IL-13 via reverse transcription quantitative polymerase chain reaction (RT-qPCR), and molecular docking studies were performed to evaluate the binding affinity of geraniol to these cytokines. Histopathological examination using hematoxylin and eosin staining was conducted on ear and dorsal skin tissues to assess eosinophil and mast cell infiltration, as well as epidermal thickness. The results demonstrated that both oral and topical geraniol significantly alleviated AD-like symptoms. Geraniol treatment led to a reduction in DLC and TLC levels in the blood, as well as downregulation of IL-4 and IL-13 expression in ear tissue. In silico studies revealed that geraniol exhibited moderate binding affinities of --4.5 kcal/mol with IL-4 and --4.9 kcal/mol with IL-13. Histopathological analysis indicated a reduction in epidermal thickness and infiltration of mast cells and eosinophils in geraniol-treated mice. In conclusion, geraniol effectively alleviated atopic dermatitis in mice by reducing clinical scores, inflammatory cell infiltration, epidermal thickening, and regional downregulation of IL-4 and IL-13 mRNA expression. *The in silico* docking studies support the hypothesis of a potential Th2-modulatory effect of geraniol.

## Introduction

Atopic dermatitis (AD), also known as atopic eczema, is a chronic inflammatory skin disorder characterized by intense itching and recurrent episodes [1]. This persistent condition is marked by inflammation, varying degrees of pruritus, and skin dryness [2]. Key features of AD include the involvement of skin folds (areas prone to friction) and the presence of itching [3].

During the acute phase, eczema manifests as a red rash accompanied by swelling, small fluid-filled blisters that may weep, and crust formation. In the chronic phase, the disorder is characterized by thickened, stiffened skin (referred to as lichenification), abrasions resulting from scratching, small raised bumps, and occasional larger nodules [4]. The hallmark of lichenification reflects the skin’s adaptive response to both epidermal inflammation and fibrotic tissue damage. A skewed T helper 2 (Th2) inflammatory response, promoted by group 2 innate lymphoid cells (ILC2s), is observed in AD; these cells, similar to eosinophils, express CD80 [5].

The clinical characteristics of AD result from interactions among immunological reactions, environmental factors, susceptibility genes, and skin barrier dysfunction [6]. A skewed immunological response favoring Th2 lymphocyte activity is a defining feature of AD, with a significant number of cells expressing mRNA for Th2 cytokines, such as IL-4 and IL-13, found abundantly in the inflammatory lesions associated with the condition [7].

Corticosteroids are a primary component of the anti-inflammatory treatment regime for AD [8]. In cases of severe AD, systemic corticosteroids can provide rapid efficacy as a short-term treatment for acute flare-ups. However, the potential for various adverse effects renders prolonged use of these medications inadvisable [9]. Ciclosporin A, an immunosuppressive medication, has demonstrated significant effectiveness in treating severe AD [10]. During ciclosporin treatment, regular monitoring of blood pressure and renal function is crucial, as the drug can induce renal impairment and hypertension [11]. Given the side effects associated with these therapeutic agents when used long-term, considerable attention is focused on identifying suitable alternative therapies for AD [12].

Geraniol, a monoterpene alcohol, is present in many aromatic plant-based essential oils and is characterized by its pleasant rose-like scent [13, 14]. It is widely utilized in the flavor industry and serves as a fragrance component in numerous cosmetic products [15, 16]. Over 250 aromatic plant-based essential oils, including bee balm oil, lavender oil, palmarosa oil, citronella oil, rose oil, lemongrass oil, and ginger oil, contain geraniol as a major constituent [13]. Notably, lemongrass essential oil has been reported to exhibit substantial anti-inflammatory effects in mouse ear edema models [17].

Geraniol is a promising candidate for pharmaceutical applications due to its documented anti-inflammatory, anti-cancer, and antioxidant properties [15]. An experimental study in mice indicated that geraniol reduces the severity of ulcerative colitis by inhibiting the infiltration of inflammatory cells into colon tissue and by blocking the release of pro-inflammatory cytokines [18]. In a mouse model of osteoarthritis, geraniol has shown potential in suppressing the nuclear factor kappa-light-chain-enhancer of activated B cells (NF-κB) and mitogen-activated protein kinase (MAPK) signaling pathways in chondrocytes, thereby reducing inflammation and preventing cartilage degradation associated with the condition [19]. While numerous studies have reported the anti-inflammatory effects of geraniol in various diseases, data regarding its immunomodulatory and anti-inflammatory effects in 2,4-dinitrochlorobenzene (DNCB)-induced AD in mice is limited. Therefore, this study aimed to evaluate the effects of geraniol on AD by assessing changes in hematological parameters, including total leukocyte count (TLC) and differential leukocyte count (DLC), physical changes in the ear skin through AD scoring, regional mRNA expression of IL-13 and IL-4, and predicting the binding affinity of geraniol to IL-13 and IL-4.

## Materials and methods

### Experimental animals

BALB/c mice, aged between seven to eight weeks and weighing between 20 g to 35 g, were obtained from the animal facility of the University of Veterinary and Animal Health Sciences, Lahore. The mice were housed in a standard environment free from pathogens, maintained at a temperature of 25 ± 5^∘^C and a relative humidity of 50%–60%. Standard laboratory feed was provided, and the mice underwent a one-week acclimation period prior to the commencement of experiments. Ethical approval for the experiments was obtained from the Research Ethics Institutional Review Board (ORIC/LCWU/392) of LCWU, Lahore, Pakistan.

### AD induction protocol

One day prior to the experiment, the hair on the dorsal skin of the mice was shaved using a trimmer and further depilated with hair removal cream. A solution of DNCB, prepared by dissolving DNCB in acetone and olive oil in a 3:1 ratio, was used to induce AD-like skin lesions, following previously established methods [20]. To induce AD-like skin lesions and symptoms, all groups, except the control group, had their dorsal skin and ears sensitized. This involved applying 120 µL of 2% DNCB to the dorsal skin and 30 µL of 2% DNCB to the pinnae of each ear on days 0 and 3. On days 6, 9, 12, 15, and 18, the sensitized mice were subjected to challenges involving the application of 120 µL of 0.5% DNCB to the dorsal skin and 30 µL of 0.5% DNCB to the pinnae of each ear. Mice in the control group received an equal volume of the vehicle.

### Experimental design

A total of 36 healthy male mice were randomly allocated into six groups, each containing six mice.

**Figure 1.**
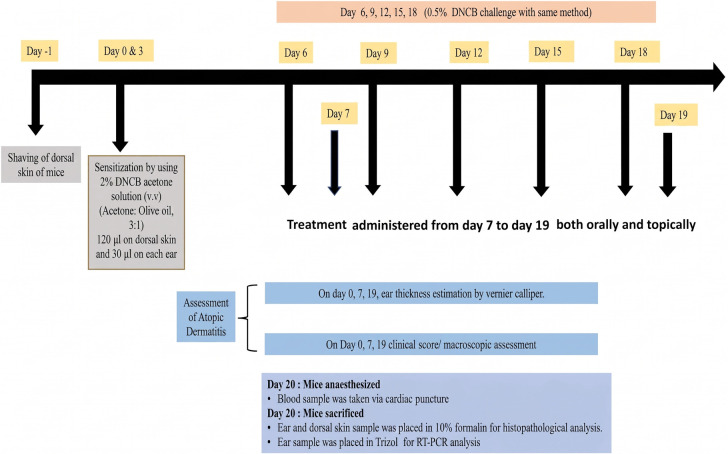
**Experimental framework for DNCB-induced atopic dermatitis and treatment administration in BALB/c mice.** Dorsal hair was removed on day --1. Mice were sensitized on days 0 and 3 by epicutaneous application of 2% DNCB (acetone:olive oil, 3:1, v/v) to shaved dorsal skin (120 µL) and both ear pinnae (30 µL/ear), followed by challenges with 0.5% DNCB on days 6, 9, 12, 15, and 18 using the same application sites. Animals were randomized into six groups (*n* ═ 6/group) and received vehicle (1% Tween 80), prednisolone (50 mg/kg; oral or topical; formulated in 10% DMSO), or geraniol (200 mg/kg; oral or topical; formulated in 1% Tween 80) from day 7–19. Clinical severity scoring and ear thickness were assessed on days 0, 7, and 19 prior to treatment administration and (on challenge days) before DNCB application. On day 20, blood was collected by cardiac puncture for leukocyte profiling, and ear and dorsal skin tissues were harvested for histopathology (10% formalin fixation) and cytokine mRNA quantification by RT-qPCR. Abbreviations: AD: Atopic dermatitis; DNCB: 2,4-Dinitrochlorobenzene; DMSO: Dimethyl sulfoxide; RT-qPCR: Reverse transcription quantitative polymerase chain reaction; mRNA: Messenger ribonucleic acid.

Group 1: Normal control group; mice underwent sensitization on days 0 and 3, followed by challenges through epicutaneous application of a mixture of acetone and olive oil (3:1) on the pinnae of both ears and on the shaved dorsal skin. 1% Tween 80 was administered orally and topically. Group 2: Diseased group; mice were sensitized with 2% DNCB on days 0 and 3, followed by five successive epicutaneous challenges from days 6–18, involving DNCB application on both ears and the shaved dorsal skin. Group 3: Standard oral prednisolone group; mice received oral prednisolone at a dosage of 50 mg/kg body weight, dissolved in a solution containing 10% dimethyl sulfoxide (DMSO) [21]. Group 4: Standard topical prednisolone group; prednisolone was topically administered at a dosage of 50 mg/kg body weight, dissolved in 10% DMSO, applied to the pinnae of both ears and the shaved dorsal skin. Group 5: Experimental oral geraniol group; mice received oral geraniol at a dosage of 200 mg/kg body weight [22], dissolved in a solution containing 1% Tween 80 [23]. Group 6: Experimental topical geraniol group; mice received a topical application of geraniol at a dosage of 200 mg/kg body weight, dissolved in a solution containing 1% Tween 80, applied to both ears and the shaved dorsal skin (Tables 1–4). All treatments were administered over 13 days, from day 7 to day 19. The experimental design is illustrated graphically in Figure 1.

**Table 1:** Experimental groups

**Group**	**Sensitization / challenge**	**Treatment administered**	**Dose**
Group 1	Acetone: Olive oil (3:1) only (no DNCB)	1% Tween 80	—
Group 2	2% DNCB on Days 0 and 3; 0.5% DNCB on Days 6, 9, 12, 15, 18	1% Tween 80	—
Group 3	Same as Group 2	Prednisolone (oral)	50 mg/kg
Group 4	Same as Group 2	Prednisolone (topical)	50 mg/kg
Group 5	Same as Group 2	Geraniol (oral)	200 mg/kg
Group 6	Same as Group 2	Geraniol (topical)	200 mg/kg

Abbreviation: DNCB: 2,4-Dinitrochlorobenzene.

**Table 2:** Specifications for topical treatments: Geraniol and prednisolone

**Treatment**	**Dose (mg/kg)**	**Dose per 30 g mouse (mg)**	**Total volume per site**	**Application method**
Geraniol	200	6	7 µL (Liquid density used to convert mg to mL) dissolved in 13 µL of 1% Tween 80, total 20 µL per site	Calibrated micropipette, spread evenly, absorption allowed
Prednisolone	50	1.5	Dissolved in 20 µL of 10% DMSO	Calibrated micropipette, spread evenly, absorption allowed

Abbreviation**:** DMSO: Dimethyl sulfoxide.

**Table 3:** Timeline of experiments and treatment schedule

**Study day**	**Procedure**	**DNCB application**	**Treatment administration**
Day 0	Sensitization 1	2% DNCB (ears + shaved dorsal skin)	None
Day 3	Sensitization 2	2% DNCB (ears + shaved dorsal skin)	None
Day 6	Challenge 1	0.5% DNCB	-
Day 7	-	-	All treatments given
Day 8	-	-	All treatments given
Day 9	Challenge 2	0.5% DNCB	All treatments given 1 h before challenge
Day 10	-	-	All treatments given
Day 11	-	-	All treatments given
Day 12	Challenge 3	0.5% DNCB	All treatments given 1 h before challenge
Day 13	-	-	All treatments given
Day 14	-	-	All treatments given
Day 15	Challenge 4	0.5% DNCB	All treatments given 1 h before challenge
Day 16	-	-	All treatments given
Day 17	-	-	All treatments given
Day 18	Challenge 5	0.5% DNCB	All treatments given 1 h before challenge
Day 19	-	-	All treatments given

Abbreviation: DNCB: 2,4-Dinitrochlorobenzene.

**Table 4:** Specifications for topical application

**Parameter**	**Description**
Application sites	Both ears (pinnae) and shaved dorsal skin
Dorsal skin area	Approximately 1.5 cm × 1.5 cm (∼3 cm^2^)
Volume per ear	30 µL on the pinna of each ear
Volume on dorsal skin	120 µL, spread evenly
Application method	Calibrated micropipette for uniform dosing
Post-application restraint	1–2 minutes of gentle restraint to allow partial absorption
Anti-licking strategy	Individual housing for ≥30 minutes post-application

### Macroscopic assessment of AD-like skin lesions

Visual assessments included evaluations of erythema, excoriation, edema, dryness, and crusting on the surface of the ears and shaved dorsal skin for each group of mice on days 7 and 19. The severity of the AD-like skin lesions was assessed through macroscopic examination using a clinical scoring method. Each level was graded on a scale from 0–3, where 0 indicates “none,” 1 signifies “mild,” 2 corresponds to “moderate,” and 3 denotes “severe” (Table 5).

**Table 5:** Assessment schedule and timing

**Parameter Assessed**	**Day 0**	**Day 7**	**Day 19**	**Timing of assessment**
AD clinical score (ear and dorsal skin)	Not assessed	Assessed	Assessed	Performed prior to drug administration and prior to DNCB challenge on assessment days.
Ear thickness (digital vernier caliper measurement)	Assessed (baseline)	Assessed	Assessed	1. Performed prior to sensitization on day 0. 2. Performed prior to drug administration and prior to DNCB challenge on day 7 and day 19.

Abbreviations: AD: Atopic dermatitis; DNCB: 2,4-Dinitrochlorobenzene.

### Measurement of ear thickness

The thickness of both the right and left ears of the mice was measured using a digital vernier caliper on day 0 as a baseline, with subsequent assessments occurring on days 7 and 19 (Table 5).

### Effect of geraniol on physical changes induced by DNCB in mice

To investigate epidermal thickness, both ear and dorsal skin tissues were subjected to gross macroscopic evaluation.

### Euthanasia of mice and sample collection

On day 20, all mice were anesthetized and subsequently euthanized. A mixture of ketamine (100 mg/kg) and xylazine (10 mg/kg) was administered intraperitoneally to induce anesthesia [24]. Following anesthesia, blood was collected via cardiac puncture. Blood samples were used to assess TLC and DLC. Ear tissues from each group were excised for the analysis of mRNA expression levels of cytokines and histopathological parameters. The shaved dorsal skin was also removed for histopathological evaluation.

### Evaluation of DLC and TLC

Blood was obtained through cardiac puncture on day 20, utilizing EDTA tubes for sample collection. TLC and DLC were analyzed using an automatic hematology analyzer.

### Assessment of IL-4 and IL-13 mRNA expression levels via real-time reverse transcription polymerase chain reaction (RT-qPCR)

In this study, ear tissues from mice were utilized to evaluate mRNA expression levels of IL-4 and IL-13 through RT-qPCR. The quantification process for mRNA expression levels followed previously established protocols [25, 26]. Total RNA was extracted using the TRIzol method, and a Nanodrop Spectrophotometer was employed to quantify the extracted RNA. Reverse transcription was conducted to convert RNA into cDNA, following the procedures outlined in the Thermo Scientific Revert Aid First Strand cDNA Synthesis Kit #K1622.

Real-time PCR was performed to amplify the cDNA. Each PCR tube was prepared with 2 µL of cDNA, 1 µL each of forward and reverse primers, and 6 µL of master mix, bringing the total volume to 12 µL with the addition of 2 µL of RNase-free water. The thermal cycler was programmed for 40 cycles, consisting of denaturation at 95^∘^C for 10 s, annealing at 58^∘^C for 20 s, and extension at 70^∘^C for 30 s. IL-4 and IL-13 expression levels were calculated using Ct values. The sequences for the forward and reverse primers were obtained from previously published studies [20, 27] (Table 6). Relative mRNA expression was quantified using the ΔCt method, with glyceraldehyde-3-phosphate dehydrogenase (GAPDH) as the reference gene, as recommended in the Bio-Rad real-time PCR application guide. Values were normalized to the mean of the normal control group (set to 1) to determine fold change. No-template controls (NTCs) were included to ensure the absence of contamination. Six biological replicates were used, each measured in two technical replicates, with the average of the technical replicates utilized for analysis.

**Table 6:** Primer sequences

**Primer**	**Forward**	**Reverse**	**Reference**
IL-4	TCACTGACGGCACAGAGCTA	CCTTCTCCTGTGACCTCGTT	27
IL-13	CCTGGCTCTTGCTTGCCTT	GGTCTTGTGTGATGTTGCTCA	20
GAPDH	ATGTGTCCGTCGTGGATCTGA	ATGCCTGCTTCACCACCTTCT	20

Abbreviation: GAPDH: Glyceraldehyde-3-phosphate dehydrogenase.

### Analysis of histopathological parameters

The ear and dorsal skin tissues were fixed in 10% neutral buffered formalin at room temperature. Following paraffin embedding, tissues were sectioned into 5 µm slices. Hematoxylin and eosin staining was performed, and a histopathologist, blinded to the experimental details, conducted the examination. Eosinophil and mast cell infiltration were assessed, alongside epidermal thickness measurements.

### Molecular Docking

Geraniol was docked against IL-4 (PDB: 5FHC) and IL-13 (PDB: 5L6Y) using AutoDock Vina. Both receptors and ligands were prepared in pdbqt format according to standard protocols. A blind docking grid of 40 × 40 × 40 Å was employed for both proteins (IL-4 grid center: –10.107, –64.905, 182.296; IL-13 grid center: –0.149, 0.014, –0.025). Exhaustiveness was set to 8, and top poses were ranked based on binding affinity (kcal/mol).

### Ethical statement

Ethical approval for the experiments was obtained from the Research Ethics Institutional Review Board (ORIC/LCWU/392) of LCWU, Lahore, Pakistan.

### Statistical analysis

Data were analyzed using GraphPad Prism version 8. Results are presented as mean ± standard deviation. A two-way repeated-measures analysis of variance (ANOVA) employing a mixed-effects model, followed by Tukey’s test, was utilized to evaluate the AD scores for both the ear and dorsal skin, in addition to measuring ear thickness. Longitudinal outcomes were analyzed using a two-way repeated-measures ANOVA (mixed-effects model), given that measurements were obtained from the same animals over time. This approach facilitated the evaluation of treatment effects, time effects, and their interaction within a single analytical framework. Model assumptions, including normality and homogeneity of variance, were assessed prior to analysis, confirming that the results were normally distributed and not heavily skewed, thereby ensuring the reliability of the ANOVA results. For comparisons between oral and topical treatments, effect estimates with corresponding 95% confidence intervals were provided alongside *P*-values to indicate the range of plausible differences between groups. The Kruskal-Wallis test followed by Dunn’s multiple comparison test was utilized for the evaluation of DLC. One-way ANOVA followed by Tukey’s test was applied for TLC and RT-qPCR analyses. A *P*-value of ≤ 0.05 was considered statistically significant.

## Results

### Impact of geraniol on AD clinical scoring

One of the primary outcomes of this study was the assessment of the clinical AD score of the ear and dorsal skin on days 7 and 19. For the AD clinical score of the ear, two-way repeated-measures ANOVA (mixed-effects model) revealed a significant main effect of the treatment group (*P* < 0.0001), while neither the effect of time (*P* ═ 0.4966) nor the group × time interaction (*P* ═ 0.1307) was significant, indicating consistent treatment differences across both time points.

On day 7, DNCB-treated mice exhibited mild AD-like lesions compared to the control group.

By day 19, the ear AD score was significantly elevated in the diseased group (2.33 ± 0.51) compared to the control (0.00 ± 0.00). Both oral (1.67 ± 0.41; *P* < 0.01) and topical (1.00 ± 0.63; *P* < 0.001) geraniol treatments significantly reduced AD scores compared to the diseased group (*P* < 0.05). Prednisolone treatment also significantly decreased ear lesion severity (1.33 ± 0.51; *P* < 0.01 vs. diseased group).

The AD clinical score of the dorsal skin was similarly evaluated on days 7 and 19. Two-way repeated-measures ANOVA (mixed-effects model) indicated a significant main effect of the treatment group (*P* < 0.0001), while neither the effect of time (*P* ═ 0.3312) nor the group × time interaction (*P* ═ 0.1634) was significant, reflecting consistent treatment differences across both time points.

On day 7, DNCB-treated mice again developed mild AD-like lesions compared to the control group.

**Figure 2.**
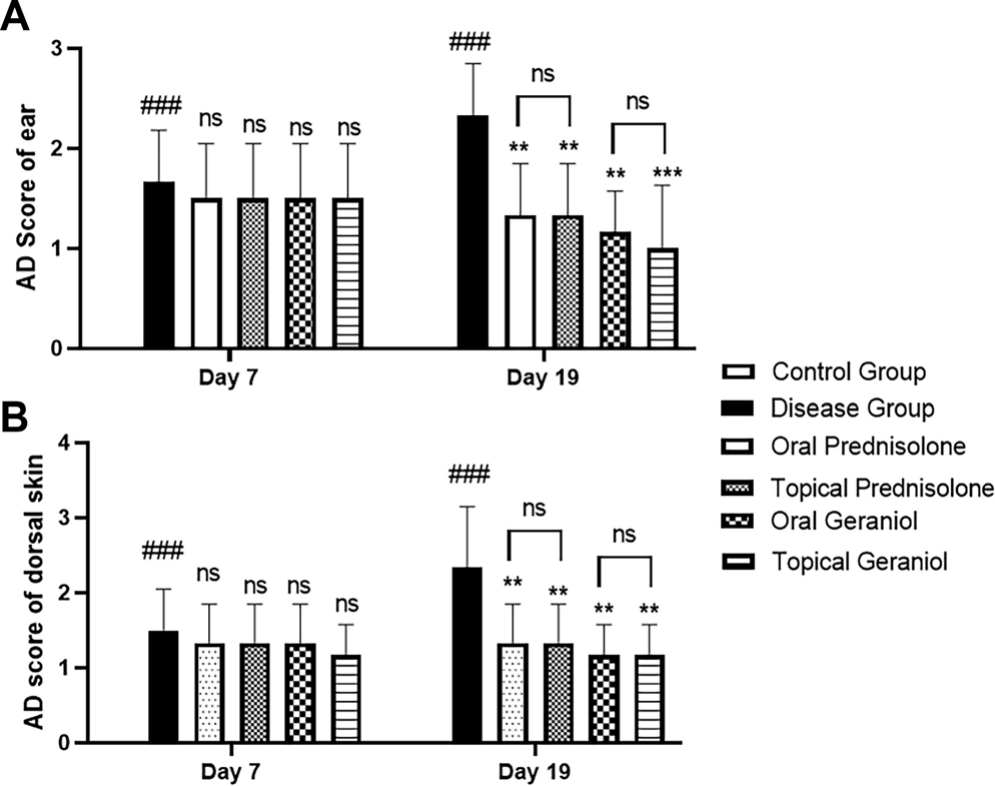
**Geraniol reduces the macroscopic severity of DNCB-induced atopic dermatitis in mice.** (A) AD clinical score of the ear and (B) AD clinical score of the shaved dorsal skin assessed on days 7 and 19 across experimental groups (control, DNCB-diseased, oral/topical prednisolone, oral/topical geraniol). Control mice received vehicle only and were not exposed to DNCB (score = 0; bars may not be visible). Data are presented as mean ± SD (*n* ═ 6/group). Statistical analysis was performed using two-way repeated-measures ANOVA (mixed-effects model) followed by Tukey’s post hoc test. ### *P ≤* 0.001 vs control; * *P ≤* 0.05, ** *P ≤* 0.01, *** *P ≤* 0.001 vs DNCB-diseased group. Brackets denote direct oral vs topical comparisons within the same treatment at the indicated time point; ns, not significant. Abbreviations: AD: Atopic dermatitis; DNCB: 2,4-Dinitrochlorobenzene; SD: Standard deviation; ANOVA: Analysis of variance; ns: Not significant.

By day 19, the dorsal skin AD score was significantly elevated in the diseased group (2.33 ± 0.81) compared to the control (0.00 ± 0.00). Both oral (1.67 ± 0.41; *P* < 0.01) and topical (1.67 ± 0.41; *P* < 0.01) geraniol treatments significantly reduced AD scores relative to the diseased group (*P* < 0.05). Prednisolone treatment also significantly decreased dorsal skin lesion severity (1.33 ± 0.51; *P* < 0.01 vs. diseased group) (Figure 2).

### Ameliorative effect of geraniol on ear thickness

**Figure 3.**
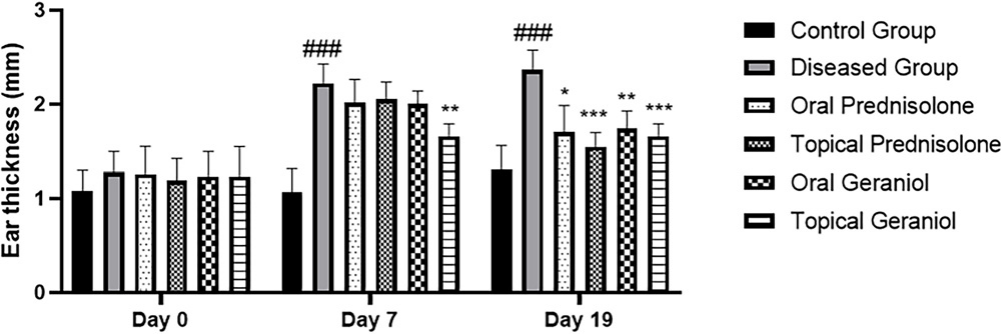
**Geraniol attenuates DNCB-induced ear swelling in mice over time.** Ear thickness (mm) was measured using a digital vernier caliper at baseline (day 0) and on days 7 and 19 following DNCB sensitization/challenge in control, DNCB-diseased, prednisolone-treated (oral/topical), and geraniol-treated (oral/topical) groups. Data are presented as mean ± SD (*n* ═ 6/group). Statistical analysis was performed using two-way repeated-measures ANOVA (mixed-effects model) followed by Tukey’s post hoc test. ### *P ≤* 0.001 vs control; * *P ≤* 0.05, ** *P ≤* 0.01, *** *P ≤* 0.001 vs DNCB-diseased group at the indicated time point. Abbreviations: DNCB: 2,4-Dinitrochlorobenzene; SD: Standard deviation; ANOVA: Analysis of variance.

Another primary outcome of this study was the measurement of ear thickness (mm) evaluated on days 0, 7, and 19. All animals were clinically normal at baseline (day 0). Two-way repeated-measures ANOVA revealed significant effects of time (F (1.715, 51.57) ═ 93.94; *P* < 0.0001), treatment group (F (5, 30) ═ 18.23; *P* < 0.0001), and a significant group × time interaction (F (10, 60) ═ 7.104; *P* < 0.0001). The significant interaction indicates that changes in ear lesion severity over time differed among experimental groups. On day 7, DNCB-treated mice exhibited mild AD-like lesions compared to control animals. By day 19, ear thickness was markedly elevated in the diseased group (2.36 ± 0.21; *P* < 0.001; 95% CI --1.541 to --0.5853) relative to control (0.00 ± 0.00). Both oral (1.74 ± 0.19; *P* < 0.01; 95% CI 0.2198–1.027) and topical (1.67 ± 0.13; *P* < 0.001; 95% CI 0.3345–1.069) geraniol treatments significantly reduced ear AD scores compared to the diseased group. Oral (1.70 ± 0.28; *P* < 0.05; 95% CI 0.1542–1.172) and topical (1.55 ± 0.15; *P* < 0.001; 95% CI 0.4400–1.193) prednisolone treatment similarly attenuated lesion severity (Figure 3A–3C).

A direct comparison of oral vs topical treatments on day 19 is presented in Table 7.

**Table 7:** Comparison of direct oral and topical administration for key endpoints

**Endpoint (day 19)**	**Oral treatment (mean ± SD)**	**Topical treatment (mean ± SD)**	***P* value**	**95% CI**
Ear AD score	1.67 ± 0.41	1.67 ± 0.41	0.99	--0.66 to 0.99
Dorsal skin AD score	1.67 ± 0.41	1.67 ± 0.41	>0.99	--0.82 to 0.82
Ear thickness (mm)	1.74 ± 0.19	1.67 ± 0.13	0.95	--0.25 to 0.41
TLC (10ˆ3/µL)	4.78 ± 0.55	5.70 ± 0.32	0.036	--1.79 to --0.04

Abbreviations: AD: Atopic dermatitis; SD: Standard deviation; CI: Confidence interval; TLC: Total leukocyte count.

### Impact of geraniol on physical changes triggered by DNCB in mice

**Figure 4.**
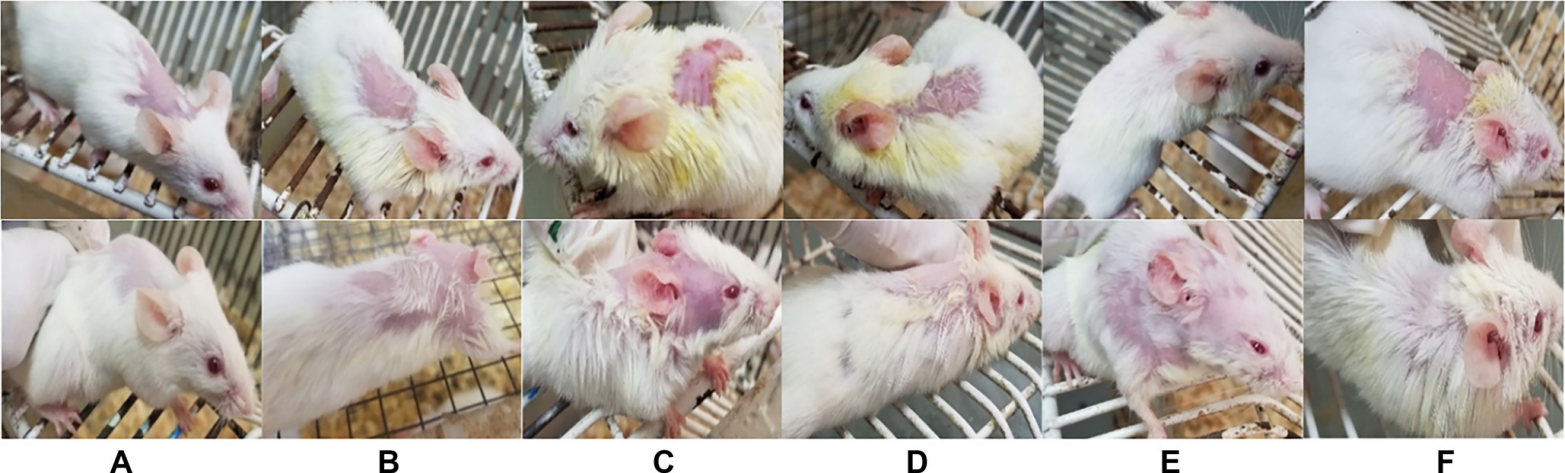
**Representative gross photographs of ear pinnae and shaved dorsal skin in the DNCB-induced AD mouse model.** Images were captured on day 7 (upper row) and day 19 (lower row) to document macroscopic changes at the application sites. Panels show: (A) vehicle control, (B) DNCB-diseased, (C) prednisolone (oral), (D) prednisolone (topical), (E) geraniol (oral), and (F) geraniol (topical). Compared with controls, DNCB exposure produced visible dermatitis characterized by erythema, edema, excoriation/dryness, and apparent skin thickening, whereas both prednisolone and geraniol treatment reduced lesion severity by day 19. Abbreviations: AD: Atopic dermatitis; DNCB: 2,4-Dinitrochlorobenzene.

To further investigate epidermal thickness, ear and dorsal skin tissues were evaluated through gross macroscopic assessment. A significant increase in epidermal thickness was observed in the diseased group compared to the normal control group. The administration of geraniol, both orally and topically, resulted in a substantial reduction in epidermal thickness in comparison to the diseased group. Figure 4 illustrates the gross macroscopic alterations in the skin and ears of mice across all groups.

### Inhibitory effect of geraniol on blood cell count

#### Total leukocyte count

**Figure 5.**
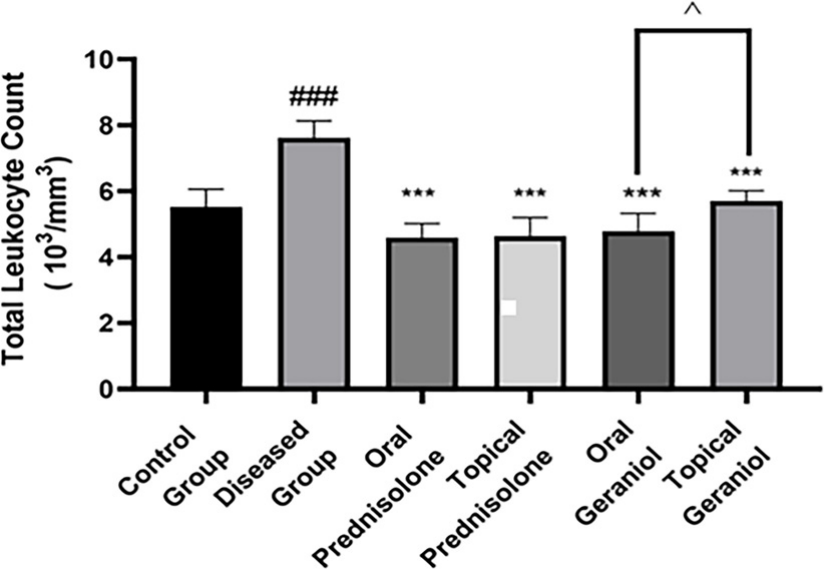
**Geraniol lowers total leukocyte count in the DNCB-induced atopic dermatitis mouse model.** Total leukocyte count (TLC; 10^3^ cells/mm^3^) was quantified in peripheral blood collected on day 20 from control, DNCB-diseased, prednisolone-treated (oral/topical), and geraniol-treated (oral/topical) groups. Data are presented as mean ± SD (*n* ═ 6/group). Statistical analysis was performed using one-way ANOVA followed by Tukey’s multiple-comparisons test. ### *P ≤* 0.001 vs control; *** *P ≤* 0.001 vs DNCB-diseased group. The bracket denotes the direct comparison between oral and topical geraniol; ˆ *P ≤* 0.05. Abbreviations: DNCB: 2,4-Dinitrochlorobenzene; TLC: Total leukocyte count; SD: Standard deviation; ANOVA: Analysis of variance.

TLC is expressed as 10^3^ cells per mm^3^ of blood, as determined using an automated hematology analyzer. Sensitization and challenge with DNCB resulted in a significant increase in TLC levels in the diseased group (7.61 ± 0.52) compared to the control group (5.51 ± 0.54). Both oral (4.78 ± 0.55) and topical (5.7 ± 0.32) administration of geraniol effectively reduced TLC levels compared to the diseased group (7.61 ± 0.52). Similarly, groups treated with oral (4.58 ± 0.54) and topical (4.63 ± 0.57) prednisolone exhibited a significant decrease in TLC levels relative to the diseased group (7.61 ± 0.52). Notably, oral geraniol was more effective than topical geraniol in lowering TLC levels (*P* < 0.05) (Figure 5).

#### Differential leukocyte count

Both oral and topical geraniol interventions resulted in a significant decrease in DLC. Additionally, oral and topical prednisolone also effectively inhibited DLC levels.

#### Neutrophils

**Figure 6.**
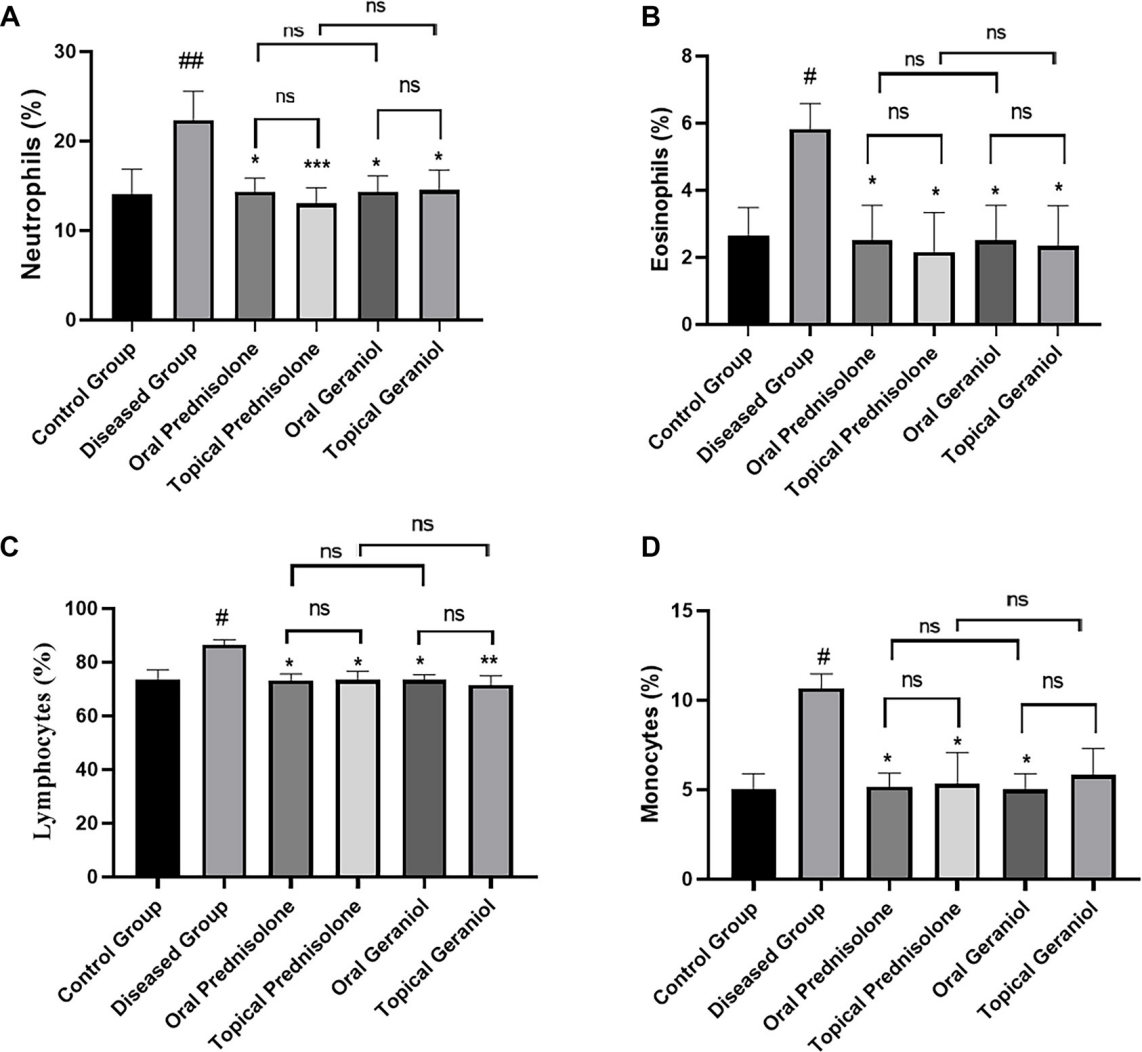
**Geraniol improves the differential leukocyte profile in the DNCB-induced atopic dermatitis mouse model.** Differential leukocyte count was determined from peripheral blood collected on day 20 and expressed as percentages of total leukocytes: (A) neutrophils, (B) eosinophils, (C) lymphocytes, and (D) monocytes in control, DNCB-diseased, prednisolone-treated (oral/topical), and geraniol-treated (oral/topical) groups. Data are presented as mean ± SD (*n* ═ 6/group). Statistical analysis was performed using the Kruskal–Wallis test followed by Dunn’s multiple-comparisons test. # *P ≤* 0.05, ## *P ≤* 0.01 vs control; * *P ≤* 0.05, ** *P ≤* 0.01, *** *P ≤* 0.001 vs DNCB-diseased group. Brackets denote pairwise comparisons; ns indicates not significant. Abbreviations: DNCB: 2,4-Dinitrochlorobenzene; SD: Standard deviation; ns: Not significant.

Sensitization and challenge with DNCB led to a marked increase in neutrophil count in the diseased group (22.33 ± 3.27%) compared to the control group (14.00 ± 2.82%). Treatment with geraniol, both orally (14.33 ± 1.51%) and topically (14.50 ± 2.26%), resulted in a significant reduction in neutrophil count compared to the diseased group (22.33 ± 3.27%). Oral and topical prednisolone also decreased neutrophil counts (14.33 ± 1.51% and 13.00 ± 1.79%, respectively) (Figure 6A).

#### Eosinophils

DNCB sensitization and challenge resulted in a significant increase in eosinophil count in the diseased group (5.83 ± 0.75%) compared to the normal control group (2.67 ± 0.82%). Treatment with geraniol, both orally (2.50 ± 1.05%) and topically (2.33 ± 1.21%), led to a substantial reduction in eosinophil counts compared to the diseased group (5.83 ± 0.75%). Oral and topical prednisolone also significantly reduced eosinophil counts (2.50 ± 1.05% and 2.17 ± 1.17%, respectively) (Figure 6B).

#### Lymphocytes

DNCB sensitization and challenge caused a significant increase in lymphocyte count in the diseased group (86.50 ± 1.87%) compared to the control group (73.67 ± 3.50%). Treatment with geraniol, both orally (73.50 ± 1.87%) and topically (71.50 ± 3.45%), resulted in a significant reduction in lymphocyte count compared to the diseased group (86.50 ± 1.87%). Oral and topical prednisolone also significantly inhibited lymphocyte counts (73.17 ± 2.48% and 73.33 ± 3.27%, respectively) (Figure 6C).

#### Monocytes

DNCB sensitization and challenge led to a significant increase in monocyte count in the diseased group (10.67 ± 0.81%) compared to the control group (5.00 ± 0.89%). Treatment with geraniol, both orally (5.00 ± 0.89%) and topically (5.83 ± 1.47%), resulted in a substantial reduction in monocyte counts compared to the diseased group (10.00 ± 1.41%). Oral and topical prednisolone also effectively inhibited monocyte counts (5.17 ± 0.75% and 5.33 ± 1.75%, respectively) (Figure 6D).

### Reduction in IL-4 mRNA expression by geraniol in the ear of mice with DNCB-induced AD

**Figure 7.**
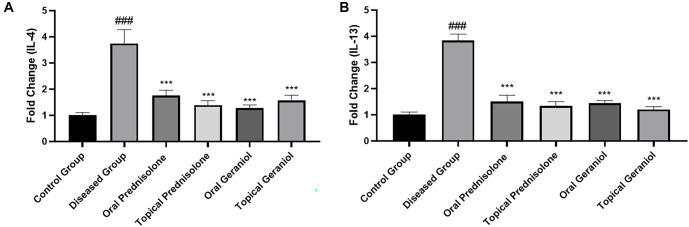
**Geraniol suppresses Th2 cytokine mRNA expression in ear tissue from DNCB-induced atopic dermatitis mice.** Relative mRNA expression (fold change) of (A) IL-4 and (B) IL-13 was quantified in ear tissue collected on day 20 using RT-qPCR, normalized to GAPDH, and expressed relative to the control group (set to 1). Bars represent mean ± SD (*n* ═ 6/group; technical replicates averaged). Statistical analysis was performed using one-way ANOVA followed by Tukey’s multiple-comparisons test. ### *P ≤* 0.001 vs control; *** *P ≤* 0.001 vs DNCB-diseased group. Abbreviations: DNCB: 2,4-Dinitrochlorobenzene; IL-4: Interleukin 4; IL-13: Interleukin 13; RT-qPCR: Reverse transcription quantitative polymerase chain reaction; mRNA: Messenger ribonucleic acid; GAPDH: Glyceraldehyde-3-phosphate dehydrogenase; SD: Standard deviation; ANOVA: Analysis of variance.

A significant increase in mRNA expression levels of IL-4 was observed in the diseased group (3.74 ± 0.52) compared to the control group (1.00 ± 0.11). Both oral (1.28 ± 0.12) and topical (1.57 ± 0.19) treatment with geraniol resulted in a noteworthy reduction in IL-4 levels compared to the diseased group (3.74 ± 0.52). Similarly, prednisolone treatment, both orally (1.76 ± 0.19) and topically (1.39 ± 0.16), also led to a significant reduction in IL-4 levels (Figure 7A).

### Reduction in IL-13 mRNA expression by geraniol in the ear of mice with DNCB-induced AD

A significant increase in mRNA expression levels of IL-13 was noted in the diseased group (3.83 ± 0.25) compared to the control group (1.00 ± 0.11). Both oral (1.45 ± 0.10) and topical (1.21 ± 0.10) treatment with geraniol resulted in a substantial reduction (*P* < 0.001) in IL-13 levels compared to the diseased group (3.83 ± 0.25). Likewise, prednisolone treatment, both orally (1.51 ± 0.24) and topically (1.34 ± 0.16), also significantly reduced IL-13 levels (*P* < 0.001) (Figure 7B).

### Impact of geraniol on histopathological changes induced by DNCB in mice

**Figure 8.**
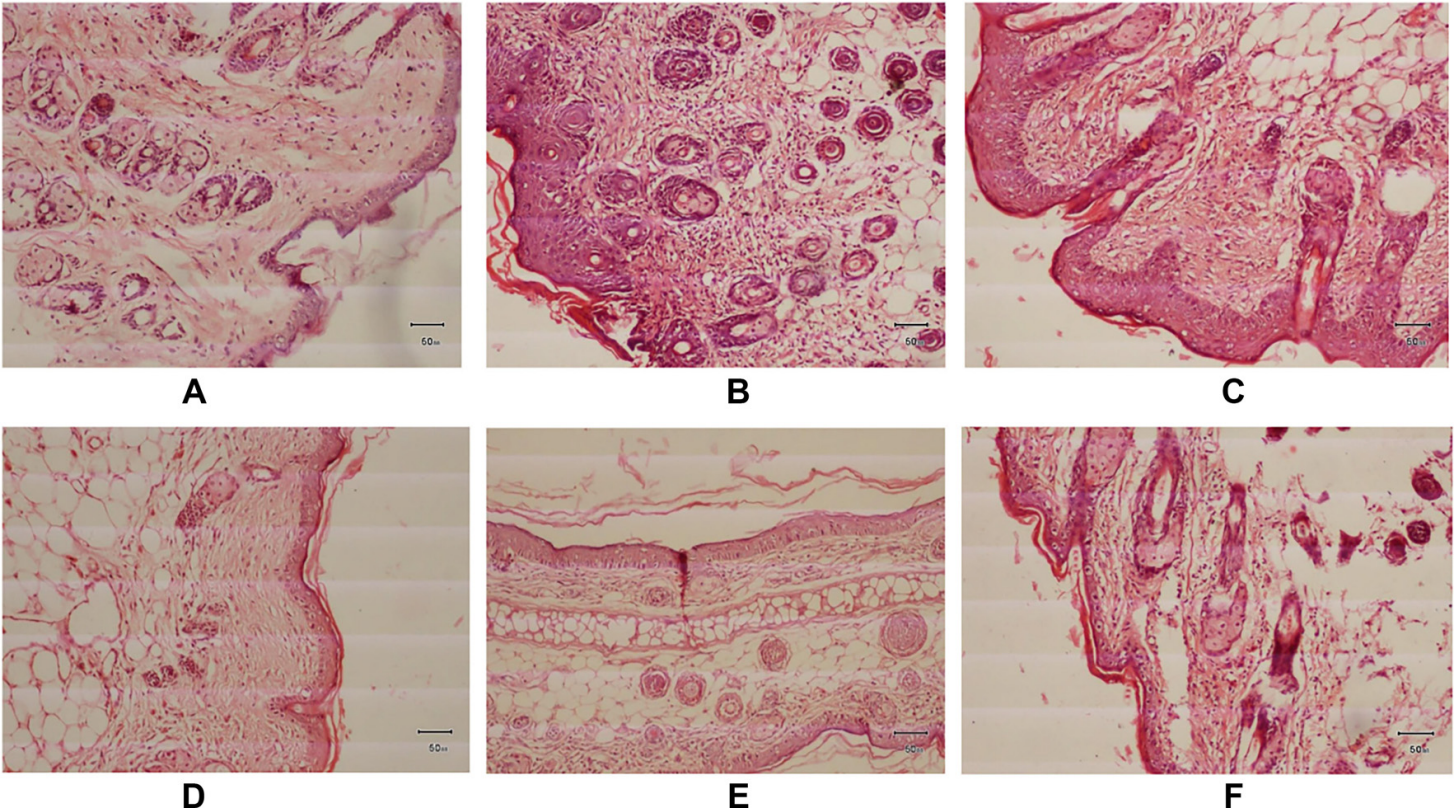
**Geraniol attenuates DNCB-induced histopathological alterations in skin.** Representative hematoxylin and eosin (H&E)–stained sections (formalin-fixed, paraffin-embedded; 5 µm) collected at study termination (day 20) from (A) vehicle control, (B) DNCB-diseased, (C) prednisolone (oral), (D) prednisolone (topical), (E) geraniol (oral), and (F) geraniol (topical) groups. (A) Control tissue shows preserved architecture with no appreciable histopathological abnormalities. (B) DNCB exposure induces a moderate increase in epidermal thickness with marked inflammatory infiltration, consistent with increased eosinophils and mast cells; a small focal area of epidermal degeneration/necrosis is also evident. (C) Oral prednisolone is associated with mild residual epidermal thickening and reduced eosinophil/mast cell infiltration relative to the diseased group. (D) Topical prednisolone produces a marked reduction in epidermal thickness with a substantial decrease in eosinophil and mast cell infiltration. (E) Oral geraniol shows marked attenuation of epidermal thickening and diminished eosinophil/mast cell infiltration. (F) Topical geraniol similarly demonstrates a notable decrease in epidermal thickness and inflammatory cell infiltration compared with the diseased group. Original magnification ×100; scale bar = 50 µm. Abbreviations: DNCB: 2,4-Dinitrochlorobenzene; H&E: Hematoxylin and eosin.

To assess epidermal thickness and the infiltration of mast cells and eosinophils, ear and dorsal skin tissues were subjected to hematoxylin and eosin (H&E) staining. A significant increase in epidermal thickness was observed in the diseased group compared to the normal control group. Administration of geraniol, both orally and topically, led to a substantial decrease in epidermal thickness in contrast to the diseased group. A marked increase in mast cell and eosinophil infiltration was noted in the untreated diseased group. Geraniol treatment resulted in a significant reduction in eosinophil and mast cell infiltration induced by DNCB (Figure 8).

### Molecular Docking results

**Figure 9.**
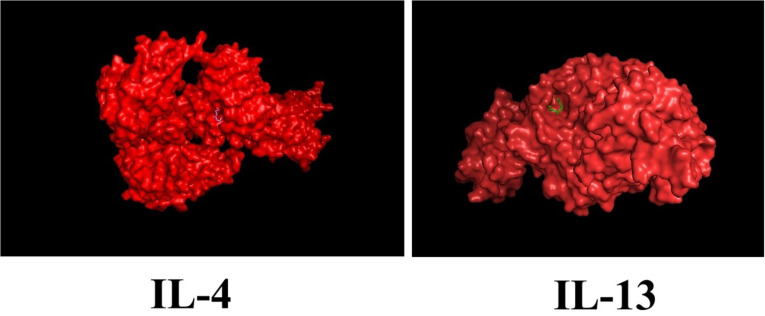
**Predicted binding of geraniol to IL-4 and IL-13 by molecular docking.** Surface representations of IL-4 (left) and IL-13 (right) showing the top-ranked docked pose of geraniol within each receptor, as generated using AutoDock Vina. The best-scoring complexes exhibited binding affinities of –4.5 kcal/mol for IL-4 and –4.9 kcal/mol for IL-13, with RMSD = 0.000 Å for the selected poses, indicating stable docked orientations. Abbreviations: IL-4: Interleukin 4; IL-13: Interleukin 13; RMSD: Root-mean-square deviation.

Geraniol demonstrated a binding affinity of –4.5 kcal/mol with IL-4 and –4.9 kcal/mol with IL-13. Lower-ranked poses showed similar energy ranges (–4.6 to –3.9 kcal/mol), with root mean square deviation (RMSD) values confirming distinct conformations. The optimal poses for both receptors exhibited an RMSD of 0.000 Å, indicating stable docked orientations (Figure 9).

## Discussion

The current study aimed to evaluate the effects of geraniol on AD by assessing hematological changes, including TLC, DLC, and neutrophil levels. Additionally, it examined physical changes to ear skin through AD scoring, regional mRNA expression of IL-13 and IL-4, and predicted the binding affinity of geraniol with these cytokines. The present study yielded novel findings, demonstrating that geraniol alleviated AD in mice by reducing clinical scores, inflammatory cell infiltration, epidermal thickening, and regional IL-4/IL-13 mRNA expression. Docking studies indicated moderate binding to IL-4 and IL-13, suggesting a potential Th2-modulatory effect.

The research focused on evaluating the anti-inflammatory and immunomodulatory effects of geraniol using a DNCB-induced mouse model of AD [28]. The induction of AD via DNCB is a widely accepted method in dermatological research [29], and the persistent increase in ear thickness, tissue edema, and epidermal hyperkeratosis observed on day 19 indicates successful AD development. Repeated DNCB application in mice induces a condition resembling AD [12]. In this investigation, DNCB administration to the ear and dorsal skin of mice triggered the emergence of AD-like skin lesions. Ear thickness serves as an established indicator of edema and inflammatory cell infiltration in the DNCB-induced model of AD. A significant decrease in AD symptoms, including redness, edema, dryness, and excoriation, was observed in the ear and dorsal skin of mice following geraniol treatment (Figure 2). Epidermal thickness was assessed physically in the ear and dorsal skin, revealing that DNCB administration resulted in moderate thickening, while geraniol treatment reduced this effect, suggesting its inhibitory impact on hyperkeratosis (Figure 5). Notably, DNCB application to both ears led to a significant increase in thickness, whereas treatment with geraniol significantly (*P* < 0.05) reduced ear thickness on days 0, 7, and 19 (Figure 3A–3C). Similar findings were observed in canines, where ilunocitinib (a JAK inhibitor) significantly reduced AD without altering hematological parameters [30]. This relief pattern in AD aligns with our findings, indicating that geraniol improves ear and skin lesions due to its anti-inflammatory effects.

Corticosteroids, antihistamines, immunosuppressants, and antibiotics are commonly used as primary therapeutic options for individuals suffering from AD [31]. There is a growing interest in discovering plant-based medications with comparable therapeutic efficacy against AD and fewer side effects [32]. Geraniol, a monoterpene alcohol, is primarily derived from aromatic plant-based essential oils, including rose, orange, lemongrass, ginger, and coriander oils [18, 33]. It exhibits a broad spectrum of physiological properties, encompassing anti-inflammatory, antioxidant, anticancer, antiulcer, and antifungal actions. The inflammatory symptoms of AD are elucidated by two fundamental theories: one posits dysregulation of the immune system, while the other addresses damage to the skin barrier. The immunological dysregulation theory suggests that an imbalance in T lymphocyte populations leads to AD [4], with a dominance of Th2 cells resulting in inflammation [6]. Th2 cytokines, particularly IL-4 and IL-13, have garnered attention as key players in AD [7]. The skin barrier dysfunction theory is supported by the observation that individuals with filaggrin gene mutations have a heightened susceptibility to AD [4]. Increased concentrations of IL-4 and IL-13 are associated with reduced expression of filaggrin, compromising skin barrier functionality [34]. Similar to geraniol therapy, isoquercitrin has also been shown to reduce skin lesions in AD by blocking inflammation [35]. Myricetin alleviated skin lesions in the same DNCB-induced model of AD, highlighting potential novel therapeutic agents for this condition [36]. The observed attenuation of DNCB-induced cutaneous inflammation by geraniol may be attributed to its antioxidant and anti-inflammatory capabilities [37].

Numerous studies have demonstrated that AD lesions exhibit increased mast cell counts, indicating the role of mast cells in cutaneous inflammation [38]. AD typically involves the proliferation, regional stimulation, and migration of eosinophils [39]. Histopathological assessments of ear and dorsal skin in mice indicated infiltration of mast cells and eosinophils. The present study’s data revealed that geraniol treatment resulted in a reduction of eosinophil and mast cell infiltration in mice with AD. It is widely recognized that individuals with AD exhibit stimulation of monocytes, lymphocytes, eosinophils, and neutrophils in their bloodstream [27]. Our results indicated a significant increase in TLC (Figure 5) and DLC (Figure 6) in the DNCB-induced diseased group. Notably, a decrease in DLC and TLC was observed in groups administered oral and topical geraniol (Figures 5 and 6A-6D). Isoquercitrin similarly reduces skin lesions by inhibiting cellular infiltration and hyperproliferation through cytokine blockade [35]. Given the data on ear and skin lesions in AD, along with the inhibition of cellular infiltration and hyperproliferation, we speculate that geraniol provides relief in AD, potentially through immunological pathways.

IL-4 and IL-13 are key cytokines produced by Th2 cells [40]. The modulation of IL-4 and IL-13 within skin lesions of AD is believed to significantly impact AD progression [41]. To assess whether geraniol suppresses allergic responses, we conducted RT-qPCR analysis to examine mRNA expression levels of IL-4 and IL-13 in ear tissue samples from mice. The diseased group exhibited elevated levels of IL-4 and IL-13 following DNCB stimulation (Figure 7A and 7B). Oral and topical administration of geraniol resulted in a notable reduction in IL-4 and IL-13 expression levels compared to the diseased group. The interleukin family, particularly IL-4, has been found in the plasma of patients with AD and is a contributing factor for itching [42, 43]. Interleukin 13 has been underestimated in the pathogenesis of AD [44], and many studies have identified it as a potential target for treatment [45–47]. Given geraniol’s broad spectrum of physiological properties, including anti-inflammatory and antioxidant effects [48], it was targeted to mitigate AD by inhibiting IL-13 and IL-4. The downregulation of these inflammatory cytokines corresponds with our histopathological data, which demonstrated marked reductions in epidermal thickness and diminished mast cell and eosinophil infiltration following oral and topical geraniol administration (Figure 8A and 8F). These findings suggest that geraniol may attenuate AD in mice by influencing immunological and anti-inflammatory pathways.

The docking analysis indicated that geraniol binds moderately to both IL-4 and IL-13, with a slightly higher affinity for IL-13 (–4.9 kcal/mol) compared to IL-4 (–4.5 kcal/mol), suggesting a potential interaction with cytokine-related regulatory sites. These computational findings are corroborated by our RT-qPCR data, which demonstrated significant downregulation of IL-4 and IL-13 expression following geraniol treatment. The combination of *in silico* and *in vitro* results suggests that the observed downregulation of IL-4 and IL-13 may be partially mediated by geraniol’s potential interaction with cytokine-related proteins. Collectively, these findings underscore the anti-inflammatory potential of geraniol and suggest that its modulatory effects on Th2-associated cytokines could contribute to its therapeutic value in conditions characterized by elevated IL-4 and IL-13 expression.

Overall, our results indicate no substantial differences in the effects of oral and topical geraniol administration, except for TLC findings. The present study has certain limitations, including the lack of exploration of additional dose levels for the investigated compound and the focus on only two cytokines, IL-4 and IL-13, while other cytokines also play significant roles in AD progression. We acknowledge the limitation of not including a dedicated wound-healing model or direct assessment of tissue repair markers in this study. Furthermore, a comparative molecular docking analysis of essential oil constituents as elastase inhibitors could provide additional mechanistic insights into chronic tissue remodeling and wound-healing aspects of AD. Additional studies are necessary to evaluate these parameters. A route-matched DMSO vehicle control was not included for topical prednisolone; although 10% DMSO is commonly used in preclinical studies and is not expected to affect outcomes, this design choice limits the ability to fully isolate any minor vehicle contribution. Further investigation is also needed to better understand and confirm the mechanisms by which geraniol affects various inflammatory cytokines.

## Conclusion

In summary, our findings indicate that geraniol treatment significantly mitigates DNCB-induced AD-like symptoms in a mouse model of AD. The alleviation of these symptoms is linked to the anti-inflammatory and immunomodulatory properties of geraniol. The investigation revealed that geraniol mitigates AD triggered by DNCB in mice by reducing ear thickness, AD scores of the ear and dorsal skin, TLC and DLC levels in blood, mast cell and eosinophil infiltration, epidermal thickness, and downregulating the mRNA expression levels of IL-4 and IL-13. Molecular docking studies revealed moderate binding affinities for IL-4 and IL-13, providing supporting evidence for a potential Th2-modulatory effect.

## Data Availability

All data is available in the manuscript.
